# Promoting Social Resilience for Racialized Older Immigrants in Canada: Stakeholder Engagement Project Findings

**DOI:** 10.1093/geroni/igaf122.862

**Published:** 2025-12-31

**Authors:** Lun Li

**Affiliations:** MacEwan University, Edmonton, Alberta, Canada

## Abstract

The Canadian population is increasingly aging and diversifying. The proportion of racialized older immigrants among older Canadians is projected to be about 25 percent after 2030. However, Racialized older immigrants experience greater social isolation and loneliness when compared to their Canada-born counterparts. Social resilience, understood as the maintenance of positive social relationships and interactions, is essential to reduce social isolation and loneliness. Thus, this study explores the strategies to promote social resilience for racialized old immigrants in Canada. A stakeholder engagement project was conducted in September 2024, including five focus groups with Chinese, Southeast Asia, South Asia, Black and Muslim communities in a metropolitan city in western Canada. Stakeholders in each focus group included community-based racialized older immigrants, service providers to older immigrants, and scholars in immigration studies. Four main groups of strategies were identified from the focus group discussion, including 1) Empowering racialized older immigrants through various learning programs and volunteering opportunities, 2) Developing awareness of the situation of racialized older immigrants within the family, 3) Increasing community-based programs, such as intergenerational projects and neighborhood activities, to enable social interaction and participation for older immigrants, and 4) Advocate for society-level changes (e.g., inclusiveness, public fundings) to support racialized older immigrants. Findings of the project reveal the urgent need to promote social resilience for racialized older immigrants. Multiple levels of strategies were proposed and discussed, with an emphasis on resilience from the society level. The project supports the idea of “social resilience is the resilience of society.

